# E-cadherin loss in RMG-1 cells inhibits cell migration and its regulation by Rho GTPases

**DOI:** 10.1016/j.bbrep.2019.100650

**Published:** 2019-05-14

**Authors:** Misako Haraguchi, Tomoko Fukushige, Takuro Kanekura, Masayuki Ozawa

**Affiliations:** a Department of Biochemistry and Molecular Biology, Japan; b Dermatology of Graduate School of Medical and Dental Sciences, Kagoshima University, Kagoshima, Japan; c Laboratory for Cell Adhesion and Tissue Patterning, RIKEN Center for Developmental Biology. Kobe, Japan

**Keywords:** E-cadherin, CRISPR/Cas9n, Cell migration, RhoGTPse, β-catenin, Dispase

## Abstract

E-cadherin is an adherens junction protein that forms intercellular contacts in epithelial cells. Downregulation of E-cadherin is frequently observed in epithelial tumors and it is a hallmark of epithelial–mesenchymal transition (EMT). However, recent findings suggest that E-cadherin plays a more complex role in certain types of cancers. Previous studies investigating the role of E-cadherin mainly used gene-knockdown systems; therefore, we used the CRISPR/Cas9n system to develop E-cadherin-knockout (EcadKO) ovarian cancer RMG-1 cell to clarify the role of E-cadherin in RMG-1 cells. EcadKO RMG-1 cells demonstrated a complete loss of the adherens junctions and failed to form cell clusters. Cell–extracellular matrix (ECM) interactions were increased in EcadKO RMG-1 cells. Upregulation of integrin beta1 and downregulation of collagen 4 were confirmed. EcadKO RMG-1 cells showed decreased β-catenin levels and decreased expression of its transcriptional target cyclin D1. Surprisingly, a marked decrease in the migratory ability of EcadKO RMG-1 cells was observed and the cellular response to Rho GTPase inhibitors was diminished. Thus, we demonstrated that E-cadherin in RMG-1 cells is indispensable for β-catenin expression and β-catenin mediated transcription and Rho GTPase-regulated directionally persistent cell migration.

## Highlights

• E-cadherin loss diminished the formation of cell clusters in RMG-1 cells.
• E-cadherin loss depleted β-catenin expression in RMG-1 cells.
• E-cadherin loss markedly decreased cell migration and response to RhoGTPase inhibitors during cell migration in RMG-1 cells.

## 1 Introduction

E-cadherin forms adherens junctions between epithelial cells and interacts with the intracellular cytoskeletal networks. Its loss is the hallmark of both sporadic and hereditary forms of diffuse gastric cancer [1]. E-cadherin was initially identified as only a tumor suppressor; however, recent studies have shown a far more complex role for E-cadherin [2]. Furthermore, a cellular context dependent variation in the role of E-cadherin has been reported.

Metastatic ovarian cancer cells exist mainly in the form of multicellular spheroids (MCSs). MCSs with high levels of E-cadherin have larger volumes and tight cellular connections [3]. The fact that transient silencing of E-cadherin expression in ovarian cancer cells inhibits collective cell migration [4], suggests that E-cadherin plays a uniquely complex role in ovarian cancer. Therefore, we developed E-cadherin-knockout (EcadKO) RMG-1 ovarian cancer cells using the CRISPR/Cas9n system [5,6] to understand the complex role of E-cadherin.

E-cadherin–mediated cell–cell adhesion and cell–extracellular matrix (ECM) interactions have been extensively studied [7,8]. For example, it has been reported that E-cadherin loss increases the adhesion of human keratinocytes to laminin and collagen [9]. In contrast, reduced cell–ECM adhesion has been reported in E-cadherin knockout MCF10A (MCF10A *CDH*–*/*–) cells (1), suggesting that the effect of E-cadherin loss on cell–ECM interactions is cell type dependent.

E-cadherin interacts with the actin cytoskeleton through the interaction with β-catenin [10]. In addition to its critical role in cellular adhesion, β-catenin functions in the Wnt signaling pathway. Downregulation of E-cadherin expression, accumulation of β-catenin in the nucleus, and activation of β-catenin./Tcf (T-cell factor) dependent transcription of target genes are hallmarks of invasive colon cancer [11,12]. Therefore, cadherins are considered to negatively regulate this pathway [13] by sequestering β-catenin [14]. In this context, it has become of interest to examine whether loss of E-cadherin activates β-catenin-dependent transcription in RMG-1 cells.

Loss of E-cadherin is thought to confer migratory abilities on immobile epithelial cells. However, some studies have reported that E-cadherin is required for epithelial dissemination and collective cell movement [2,4,15]. Rho GTPases play a central role in cell migration [16]. The role of E-cadherin in Rho signaling [17,18] and Rac-based direction-sensing mechanism [19] during collective cell migration have also been elucidated.

In the present study, we generated EcadKO RMG-1 cells and elucidated the role of E-cadherin in cell morphology, cell–cell and cell–substrate adhesion, β-catenin expression, β-catenin mediated gene expression, and cell migration and its regulation by Rho GTPases.

## 2 Materials and methods

### 2.1 Ethical statement

Experiments with recombinant DNA technology were performed in accordance with the guidelines of the Kagoshima University Committee on recombinant DNA. The security approval numbers are 27062 and S28026.

### 2.2 Cell lines and culture

Human ovarian mesonephroid adenocarcinoma cell line RMG-1 [20] was obtained from the Japanese Collection of Research Bioresources Cell Bank (JCRB, Osaka).

### 2.3 CRISPR/cas9n plasmid design

**Fig. 1:**
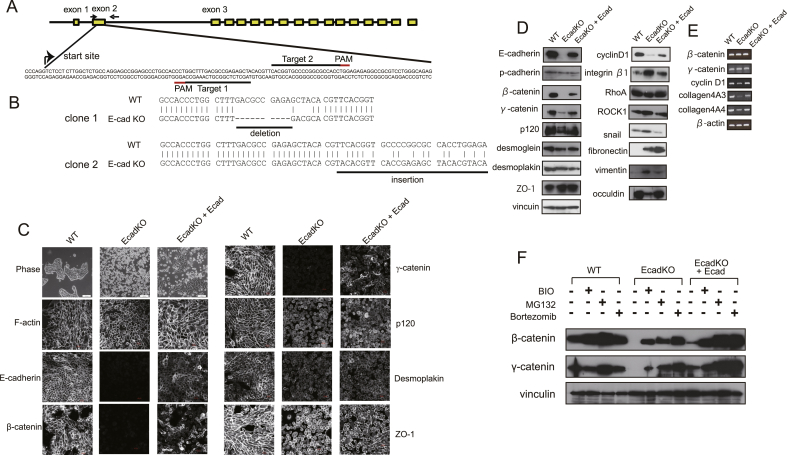
Generation of EcadKO RMG-1 cells. A, Schematic illustration of E-cadherin gene structure and sequences around the target loci. The yellow boxes indicate exons encoding the E-cadherin protein. The gRNA target sequences and protospacer adjacent motif (PAM) sequences are indicated by black and red underlining, respectively. The arrows indicate the location of PCR primers. B, The genomic sequences around the target sites of wild-type (WT) and E− EcadKO RMG-1 cells. C, Cell morphology (Phase), cytoskeletal organization (F-actin), and protein expression and localization are shown. Cell morphology were visualized using phase-contrast microscopy. Images of actin cytoskeletons stained with rhodamine X-conjugated phalloidine (F-actin) and images of immunofluorescence staining were visualized using confocal laser scanning microscope (LSM 700). D, Immunoblots analysis of indicated proteins are shown. E, Representative gel electrophoresis images of indicated genes after RT-PCR. F, Immunoblot analysis of indicated proteins. Cells were treated with 3 μM of BIO, 10 μM of MG132, and 100 nM of Bortezomib for 12 h.

To select the target sequence for genome editing, we used the CRISPR Design Tool (http://tools.genome-engineering.org). Two target sites were selected (Fig. 1A). The oligonucleotides used to construct guide RNAs (gRNAs) for the human E-cadherin gene were: g Ecad 1 (5′- caccgTAGCTCTCGGCGTCAAAGCC-3′), g Ecad 2 (5′-caccgCACGGTGCCCCGGCGCCACC-3′).

These oligonucleotides were cloned into the guide RNA expression vector pX335-U6-Chimeric_BB-CBh-hSpCas9n (D10A). These constructs were designated pX335-Cas9-g Ecad 1 and pX335-Cas9-g Ecad 2. pX335-U6-Chimeric_BB-CBh-hSpCas9n (D10A) was a gift from Dr. Feng Zhang.

### 2.4 CRISPR/cas9n-mediated engineering of the RMG1 cell genome

RMG1 cells were co-transfected with pX335-Cas9-g Ecad 1, pX335-Cas9-g Ecad 2, and pEGFP-N1 (Clontech, Mountain View, CA). G418 was applied for selection. E-cadherin expression in the emerging colonies was assessed using immunoblotting. Genomic DNAs were isolated from the clones with no E-cadherin. DNA fragments were amplified using polymerase chain reaction (PCR). Primers 999F (5′-CCACCCCAGGTCTCCTCTTG -3′) and 1181R (5′- TTCTCGGCCCCTTTCCAACC-3′) were used and the PCR products were directly sequenced.

### 2.5 Transfection of EcadKO cells with mouse E-cadherin cDNA (rescue experiment)

EcadKO cells were transfected with a HA-tagged mouse E-cadherin plasmid (pCAGGS-E-cadherin HA) and pcDNA6/TR using Screen Fect A plus (Wako, Osaka, Japan). Blasticidin was used to select transfected clones.

### 2.6 Immunoblotting

Immunoblotting of total cell lysate, including the nuclear fraction, was performed as described previously, with minor modifications [21].

### 2.7 Immunofluorescence staining

Immunofluorescence staining was performed as described previously, with minor modifications [5].

### 2.8 RNA isolation and RT-PCR analysis

RNA isolation and RT-PCR were performed as described previously [5] using the primers listed in Table 1.

**Table 1:** Primer sequences for RT-PCR.

*Gene name*	oligonucleotides
β-catenin	5′- GAGGGTACGAGCTGCTATGT-3’
	5′- CGTGTCTGGAAGCTTCCTTT-3′
γ-catenin	5′- CTACGACTCGGGTATCCACT-3′
	5′- CCTTCTTCGACAGCTGGTTC-3′
*Cyclin D1*	5′-TGGAGGTCTGCGAGGAACAG-3′
	5′- AGGAGCAGCTCCATTTGCAG-3′
*Occuldin*	5′-GGATCCTGTCTATGCTCATT-3′
	5′-ACTGGTAACAAAGATCACCA-3′
*Snail*	5′- ATGCCGCGCTCTTTCCTCGT-3
	5′- GCCTTTCCCACTGTCCTCATC-3′
*vimentin*	5′- AATGGCTCGTCACCTTCGTGAAT-3′
	5′- CAGATTAGTTTCCCTCAGGTTCAG-3′
*COL4A3*	5′- ACGGACTTGTCGGTGTACCA-3′
	5′- TTGCCCTTGACTCCCTCACT-3′
*COL4A4*	5′- GGCCCAGATAACAGAACGGA-3′
	5′- GACCTGGTGAACCAGCACAA-3′
*β-actin*	5′- CAAAGACCTGTACGCCAACAC-3′
	5′- CATACTCCTGCTTGCTGATCC-3′

### 2.9 Cell proliferation assay

Cell proliferation assay was performed according to manufacturer's instructions (Cell proliferation reagents DOJINDO, Kumamoto, Japan).

### 2.10 Dissociation assay

The dissociation assay was performed as described previously, with minor modifications [22]. Briefly, the number of particles per field (Np) as well as the total number of cells (Nc) were counted. The particles were incubated for 10 min in the presence of 5 mM ethylene glycol bis [b-aminoethylether] N, N, N′, N′-tetraacetic acid (EGTA) and they were re-exposed to mechanical stress. The extent of cell dissociation was represented by the index Np/Nc.

### 2.11 *Cell adhesion assa*y

The cell adhesion assay was performed as described previously, with minor modifications [21]. Briefly, cells were seeded in 96-well plates coated with 10 μg/mL of collagen I, 10 μg/mL of vitronectin, and 2 μg/mL of laminin 5 followed by 10–20 min of incubation. The number of attached cells was expressed as the ratio of the number of cells adhering to the plates during 10–20 min incubation and the number of adhered cells after 2 h of incubation in the presence of fetal calf serum.

### 2.12 Detachment assay

The detachment assay was performed as described previously, with minor modifications [21]. Briefly, cells were dissociated from the culture plate by incubation with 2.4 U/mLof dispase at 37 °C for 20 min. The data are presented as a percentage ratio of remaining adherent cells to untreated cells.

### 2.13 Cell migration assay

Cell migration assays were performed as described previously [23]. At the indicated time, phase－contrast microscopy imaging was performed (five images per cell) to measure the gap length between cell layers (at least three points per image). For inhibition studies, 125 μM of the Rac inhibitor NSC23766 was added to each well and the cells were incubated for 30 min before removing the silicon inserts [23]. During the cell migration assay, 2.5 μM of NSC23766 or 10 μM of ROCK inhibitor Y27632 were added to culture medium.

### 2.14 Statistical analyses

The Student *t*-test was used to perform statistical analyses of independent samples. Data are expressed as the mean ± SEM. Depending on the experiment, all P < 0.05 or P < 0.01 were considered statistically significant (**P < 0.05 and *P < 0.01).

## 3 Results

### 3.1 The generation of EcadKO and EcadKO + Ecad cells in RMG-1 cells

To determine the precise role of E-cadherin in ovarian cancer, we used the ovarian cancer cell line RMG-1 to develop EcadKO RMG-1 cells using the CRISPR/Cas9n system [6] (Fig. 1A). Two clones with CRISPR/Cas9n mediated E-cadherin deletion were selected. One clone showed a 10-bp deletion and 4-bp mutation (clone 1). The other clone showed a 62–bp insertion (clone 2) (Fig. 1B). In subsequent experiments, we primarily used clone 1; however, similar results were obtained with clone 2. Immunofluorescence and immunoblotting confirmed the absence of E-cadherin (Fig. 1C and D). To confirm the role of E-cadherin in RMG-1 cells, we prepared rescue cells that restored the expression of wild-type mouse E-cadherin in EcadKO cells and designated it as EcadKO + Ecad cells.

### 3.2 Ablation of the E-cadherin gene alters cellular morphology and protein expression in RMG-1 cells

EcadKO RMG-1 cells showed a more rounded morphology and existed individually instead of forming cell clusters (Fig. 1C, Phase). In contrast, EcadKO + Ecad cells exhibited cell–cell adherence and formed clusters. To directly observe the effect of E-cadherin ablation on the cytoskeleton of RMG-1 cells, we examined the localization of actin cytoskeleton using immunofluorescence staining for F-actin. EcadKO RMG-1 cells revealed cortical actin and had very few stress fibers compared with the wild-type (WT) and EcadKO + Ecad cells (Fig. 1C, F-actin). β-Catenin expression was depleted in EcadKO RMG-1 cells whereas the EcadKO + Ecad cells showed partially rescued expression. The expression of γ-catenin was also mostly depleted. Furthermore, the expressions of p120 decreased in the EcadKO cells (Fig. 1C and D). Desmoplakin expression at the desmosome was reduced in EcadKO RMG-1 cells (Fig. 1C); however, the total protein levels remained unchanged (Fig. 1D). ZO-1 expression at tight junctions remained the same (Fig. 1C and D). Because E-cadherin expression at the cell–cell junctions of EcadKO + Ecad cells is lower than that of WT cells, EcadKO + Ecad cells partially rescued β-catenin and γ-catenin expression of E-cadKO RMG-1 cells (Fig. 1C and D).

### 3.3 Loss of E-cadherin allowed β-catenin and γ-catenin degradation and impaired β-catenin-dependent transcription in RMG-1 cells

Most E-cadherin deficient cells retain β-catenin expression [24,25]. In contrast, EcadKO RMG-1 cells demonstrated β-catenin depletion and significant γ-catenin reduction (Fig. 1C and D), irrespective of the presence of alternative binding partners such as P-cadherin and desmoglein [26] (Fig. 1D). Because β-catenin and γ-catenin transcription levels were unchanged in EcadKO RMG-1 cells (Fig. 1E), we believe that the reduction in both protein levels was attributable to protein degradation. In the absence of a Wnt signal, non-junctional β-catenin is rapidly phosphorylated by GSK-3β in the complex and is degraded by the proteasome system [26]. Therefore, we examined the effect of BIO, a GSK-3β inhibitor, and of MG132 and bortezomib, both proteasome inhibitors, on β-catenin and γ-catenin expressions. Both protein levels significantly increased in EcadKO RMG-1 cells because of the treatment with the GSK-3β and proteasome inhibitors (Fig. 1F). E-cadherin was originally considered to prevent β-catenin mediated transactivation [27]. However, in EcadKO RMG-1 cells, the expression of cyclinD1, a target gene of β-catenin dependent transcription, was reduced (Fig. 1D and E).

### 3.4 Loss of E-cadherin enhanced cell dissociation in RMG-1 cells

**Fig. 2:**
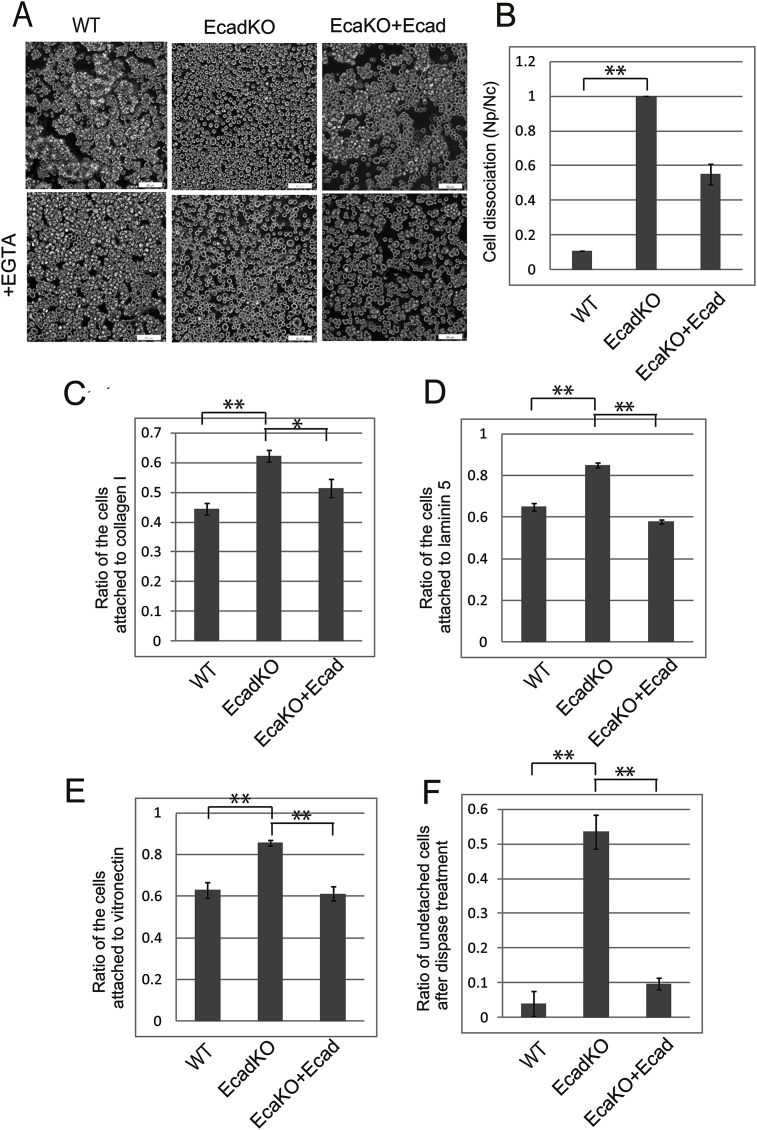
E-cadherin loss increased cellular dissociation, and increased cell–substrate adhesion. A, Representative image of cell dissociation. Images of cells after exposure to mechanical stress by pipetting after substrate detachment (upper). Images of cells exposed to mechanical stress by pipetting after EGTA treatment (lower). B, The extent of cell dissociation was represented by the Np/Nc index. Both Np and Nc were counted in minimum five different fields. C, D, E, The relative number of cells adhering to the plates coated with collagen I (C), laminin 5 (D), and vitronectin (E) are indicated. F, Ratios of undetached cells after dispase treatment. Values are expressed as the mean ± SEM of five images per sample. Experiments were repeated three times. Statistical significance is indicated with asterisk (*P < 0.05 and **P < 0.01; Student *t*-test).

With decreased expression of cell–cell adhesion proteins in EcadKO RMG-1 cells, cell–cell interactions were expected to weaken. To confirm this, we performed a cell dissociation assay. Dispase treatment of WT cells resulted in the detachment of cells in the form of epithelial sheet. This sheet was resistant to pipetting-based mechanical dissociation and resulted in the formation of larger particles (Fig. 2A, upper panel). After EGTA treatment, these big particles dissociated into single cells (Fig. 2A, lower panel). In contrast, EcadKO RMG-1 cells detached as single cells even in the absence of mechanical dissociation (Fig. 2A, upper panel). We measured the number of particles after mechanical dissociation (Np) and the total number of cells after EGTA treatment (Nc). Indeed, EcadKO RMG-1 cells showed a drastic increase in cell dissociation (Fig. 2B).

### 3.5 Loss of E-cadherin increased cell–substrate adhesion and decreased cell–substrate detachment by dispase treatment in RMG-1 cells

The effect of cell–cell adhesion on cell–ECM adhesion has been widely studied and a cell type based variation has been observed. To investigate the role of E-cadherin in cell–ECM adhesion of RMG-1 cells, we performed an adhesion assay. The adhesion ratios of EcadKO RMG-1 cells to collagen I, laminin 5, and vitronectin were higher (Fig. 2C, D, and E) than those of WT cells. Integrin β1 is the major adhesion receptor for various ECM components such as collagen [28], laminin 5 [29], and vitronectin [30]. The expression of integrin β1 was upregulated in EcadKO RMG-1 cells (Fig. 1D). We also performed a cell detachment assay with dispase treatment. Dispase is a neutral protease that can separate intact epithelial sheets from the substratum [31]. EcadKO RMG-1 cells demonstrated significant resistance to dispase treatment. After 20 min of treatment, over half of the EcadKO RMG-1 cells remained attached, whereas most of the WT cells were detached (Fig. 2F). Dispase cleaves collagen 4 [31]. EcadKO RMG-1 cells showed reduced expressions of collagen 4A3 and collagen 4A4 (Fig. 1E).

### 3.6 Loss of E-cadherin inhibited cell migration and its regulation by rho GTPases in RMG-1 cells

**Fig. 3:**
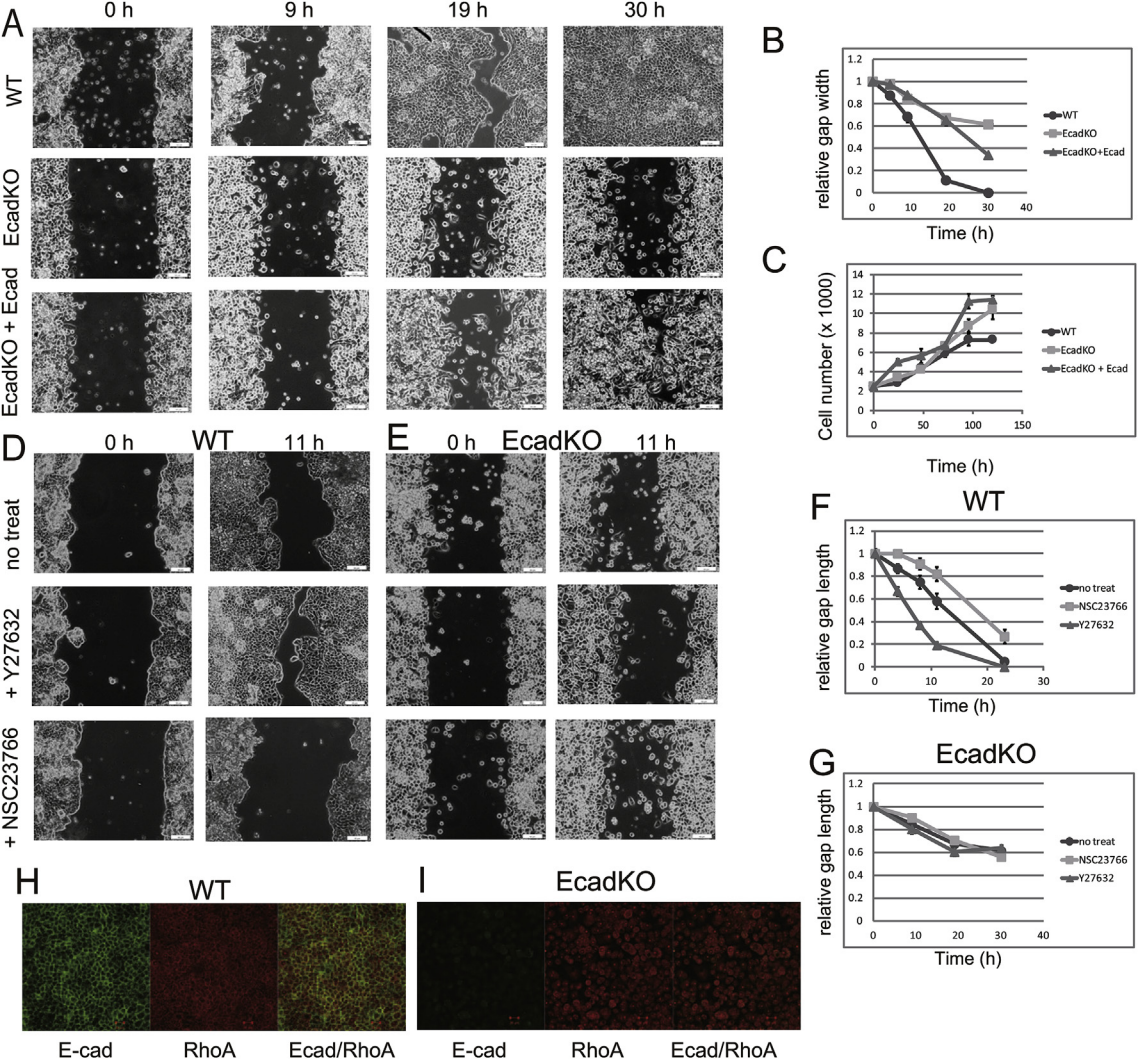
Loss of E-cadherin compromised cell migration and response to ROCK and Rac inhibitors. A, Representative images of migrating cells indicating the times after lifting of the culture insert. B, Determination of the ratios of the gap width represented by the gap width at indicated times divided by the gap width at 0 h. Gap widths are expressed as mean ± SEM of five images per sample. C, Cell growth curves. D and E, Representative images of migrating WT (D) and EcadKO RMG-1 (E) cells in the absence or presence of ROCK and Rac inhibitors. F and G, Determination of the ratios of gap width in WT (F) and EcadKO RMG-1 (G) cells. Gap widths are expressed as mean ± SEM of five images per sample. H and I, Immunofluorescence staining of WT (H) and EcadKO RMG-1 (I) cells with anti-E-cadherin and anti-RhoA antibodies were visualized using LSM 700.

Recent findings [2,4,15] have challenged the traditional role of E-cadherin in cell migration. We performed a cell migration assay to confirm the role of E-cadherin in the migration of RMG-1 cells. While WT cells demonstrated collective cell migration and close the gap in less than 30 h, EcadKO RMG-1 cells migrated individually and close only 40% of the gap in 30 h. EcadKO + Ecad cells migrated in clusters, and they close 70% of the gap (Fig. 3A and B). Because E-cadherin expression in EcadKO + Ecad cells is lower than that in WT cells, EcadKO + Ecad cells partially rescued the corrective migration of WT cells.

To rule out the possible inhibitory effect of E-cadherin loss on growth, we measured cell proliferation and confirmed that E-cadKO RMG-1 cells do not show decreased proliferation (Fig. 3C). Because Rho GTPases play a central role in all types of cell migration [16,23], we examined the involvement of the Rac and RhoA GTPases in the migration of EcadKO RMG-1 cells. Recent studies have shown that Rac is activated at the leading front of migrating cells [32]. Therefore, we investigated the effect of Rac inhibitor on cell migration. NSC 23766 delayed the migration of WT cells (Fig. 3D, F), but not that of EcadKO RMG-1 cells (Fig. 3E, G). In addition to Rac, RhoA has also been reported to be active at the leading edge of cells [32]. Rho kinase (ROCK) is an effector protein of RhoA and ROCK inhibitor Y27632 can increase cell migration [33]. Y27632 accelerated the migration of WT cells (Fig. 3D, F) but not that of EcadKO RMG-1 cells (Fig. 3E, G). These findings suggest that Rac and ROCK do not regulate cell migration in the absence of E-cadherin in RMG-1 cells. Total cellular levels of RhoA and ROCK were similar between WT cells and EcadKO RMG-1 cells (Fig. 1D). In WT cells, RhoA and E-cadherin colocalized at cell–cell junctions (Fig. 3H), whereas RhoA was found in the cytoplasm of EcadKO RMG-1 cells (Fig. 3I).

## 4 Discussion

E-cadherin is considered a negative regulator of the Wnt pathway through its sequestration of β-catenin and tumor suppressors [11]. E-cadherin has been considered to recruit β-catenin to cell membrane and prevent its nuclear localization and transactivation [27]. On the other hand, in the absence of a Wnt signal, no junctional β-catenin was considered to be degraded [26]. In EcadKO RMG-1 cells, β-catenin is completely degraded. Wnt signal might be absent in RMG-1 cells. Therefore, E-cadherin would not function as a negative regulator of β-catenin in RMG-1 cells.

E-cadherin downregulation is a hallmark of EMT and has been shown to be sufficient to induce EMT in some but not all cancer cell lines [1]. In our study, the EcadKO cells were round in shape and did not possess a fibroblastic morphology but showed increased levels of mesenchymal markers, such as snail, fibronectin, and vimentin, and reduced levels of epithelial markers, such as occuldin (Fig. 1D). This indicated that EcadKO cells revealed a partial EMT phenotype.

Both EcadKO cells and the cell in full EMT condition, showed loss of E-cadherin expression and neither showed collective cell migration. The cells in full EMT condition showed enhanced individual cell migration and migrated much faster than WT cells [5] In contrast, EcadKO cells showed inhibition of migration.

E-cadherin downregulation is commonly observed in epithelial tumors [1]. E-cadherin is considered to be a suppressor of tumor invasion and metastasis in several epithelial cancers [2]. It is a major homophilic cell–cell adhesion molecule and has been considered to inhibit cell motility [19]; however, EcadKO RMG-1 cells showed the inhibition of migration. E-cadherin knockout MCF10A (MCF10A *CDH*–*/*–) cells also showed slower migration compared to WT cells [1] E-cadherin knockout cells were generated to delete E-cadherin only. Therefore, accurate E-cadherin function should ideally be demonstrated. In contrast, E-cadherin-low tumor cells might be developed by alteration of transcriptional regulation, such as EMT, and might have altered expression of other genes showing enhanced migration and invasion.

While collective migration was observed in WT cells, individual migration was observed in the EcadKO RMG-1 cells. In addition, the time taken to close the gap was significantly longer in the EcadKO RMG-1 cells compared with the WT cells. Thus, we showed that E-cadherin is required for collective migration of RMG-1 cell. E-cadherin has been reported to play a role in generating front/back polarity during collective cell migration [19]. E-cadherin functions at the leading edge of the cells with Rac as a part of the direction-sensing mechanism. It has been reported that knocking down E-cadherin in border cells randomizes the distribution of Rac activity and results in the loss of directional persistence [19]. We hypothesized that loss of E-cadherin might randomize the distribution of Rac activity and Rac inhibitors might not have an additional effect on EcadKO RMG-1 cell migration. Another Rho GTPase, RhoA, has also been reported to be essential for directed cell migration [34]. ROCK, an effector of RhoA, has been reported as an inhibitor of cell migration [35]. In the present study, treatment with the ROCK inhibitor Y27632 significantly accelerated cell migration in WT cells, while it did not have any effect in EcadKO RMG-1 cells (Fig. 3D–G). We showed that while RhoA localizes with E-cadherin at cell–cell junctions of WT cells, it is diffused throughout the cytoplasm of EcadKO RMG-1 cells (Fig. 3H and I). This might indicate that RhoA might not have a role in the activation of ROCK at cell–cell junctions and in the induction of actomyosin-mediated retraction in EcadKO RMG-1 cells, which would also explain the lack of enhanced migration after treatment with a ROCK inhibitor. These findings indicate a role for E-cadherin in correctly orienting RhoA and Rac and regulating directionally persistent migration.

## Conflicts of interest: none

The authors have no competing interests to declare.
